# Anti-Menopausal Effect of Heat-Killed *Bifidobacterium breve* HDB7040 via Estrogen Receptor-Selective Modulation in MCF-7 Cells and Ovariectomized Rats

**DOI:** 10.4014/jmb.2402.02035

**Published:** 2024-06-17

**Authors:** Hyeon Jeong Kim, Kyung Min Kim, Min-Kyu Yun, Duseong Kim, Johann Sohn, Ji-Won Song, Seunghun Lee

**Affiliations:** Biohealthcare R&D Center, HYUNDAI BIOLAND Co., Ltd., Ansan 15407, Republic of Korea

**Keywords:** *Bifidobacterium breve*, estrogen receptor β, ovariectomy, menopause, osteoporosis

## Abstract

Menopause is induced by spontaneous ovarian failure and leads to life quality deterioration with various irritating symptoms. Hormonal treatment can alleviate these symptoms, but long-term treatment is closely associated with breast and uterine cancer, and stroke. Therefore, developing alternative therapies with novel anti-menopausal substances and improved safety is needed. In our study, heat-killed *Bifidobacterium breve* HDB7040 significantly promoted MCF-7 cell proliferation in a dose-dependent manner under estrogen-free conditions, similar to 17β-estradiol. This strain also triggered *ESR2* expression, but not *ESR1*, in MCF-7 cells. Moreover, administrating HDB7040 to ovariectomized (OVX) Sprague-Dawley (SD) female rats reduced estrogen deficiency-induced weight gain, fat mass, blood triglyceride, and total cholesterol levels. It also recovered collapsed trabecular microstructure by improving trabecular morphometric parameters (bone mineral density, bone volume per tissue volume, trabecular number, and trabecular separation) and decreasing blood alkaline phosphatase levels with no significant changes in uterine size and blood estradiol. HDB7040 also significantly regulated the expression of *Tff1*, *Pgr*, and *Esr2*, but not *Esr1* in uteri of OVX rats. Heat-killed *B. breve* HDB7040 exerts an anti-menopausal effect via the specific regulation of ERβ in vitro and in vivo, suggesting its potential as a novel substance for improving and treating menopausal syndrome.

## Introduction

Menopause is the condition of permanent amenorrhea induced by spontaneous ovarian failure, signifying the loss of reproductive capacity in women [1, 2]. It is accompanied by various physical and psychological symptoms, such as vasomotor symptoms (*e.g.*, night sweats and hot flashes), genital dryness, weight gain, depression and osteoporosis, thereby reducing the life quality of women [1, 2]. Although hormone replacement therapy (HRT) is the most effective and well-known approach to treat various menopausal symptoms, it can also increase the incidence of various diseases, including breast cancer, cardiac problems, and thromboembolism [3, 4].

Estrogen receptors (ERs) are members of the nuclear hormone receptor superfamily of transcription regulators that directly and indirectly regulate estrogen signaling pathways [5]. Two ER subtypes are found in humans: ER alpha (ERα) and ER beta (ERβ). They function as key transcriptional mediators in various pivotal organ systems and tissues, including the central nervous, skeletal and immune systems, uterus, ovary, and testis [6]. HRT mainly targets ERs since it regulates estrogen pathways; however, ERs can inevitably cause HRT-related diseases. ERα is a critical factor for the occurrence of ER-positive breast cancer, which accounts for approximately 70% of all breast cancers [6, 7].

Probiotics are non-pathogenic and health-beneficial live microorganisms. The main representatives are *Lactobacillus*, *Bifidobacterium*, *Streptococcus*, *Bacillus*, and *Enterococcus* [8, 9]. Probiotics are effective in immunomodulation, maintaining the gastrointestinal environment, and improving various diseases; however, the practical applications of probiotics are limited in safety and storage stability [8, 9]. Heat-killed probiotics have emerged as an alternative type of probiotic, without the aforementioned limitations, as they offer longer shelf life and ease of distribution/storage than animated probiotics [8, 9]. Generally, heat-killed probiotics contain bacterial cell wall components (exopolysaccharides, peptidoglycans, and lipoteichoic acids), cytoplasmic ingredients (DNA and intracellular enzymes), and bacterial metabolites. These components are crucial in immunomodulation and maintaining homeostasis in the host by communicating with the host’s gut [8]. Inanimate probiotics, including *L. reuteri*, *L. casei*, *L. plantarum*, *L. paracasei*, *E. faecium*, *B. breve*, *B. longum*, and *B. bifidum*, exert various pharmaceutical effects, such as immunomodulation, anti-obesity, anti-bacterial, anti-cancer, antioxidant, and anti-osteoporotic activities [8-13]. Nevertheless, the underlying mechanism of action of heat-killed probiotics against ERs has been rarely explored, and developing novel anti-menopausal substances with low safety risks is needed.

Hence, with the aim of mitigating menopausal symptoms, we evaluated the regulatory effect of heat-killed *B. breve* HDB7040 on ER signaling pathways in the ER-positive human breast adenocarcinoma MCF-7 cell line and ovariectomized rats.

## Materials and Methods

### Materials

The RPMI1640 medium was purchased from Welgene (Republic of Korea). Fetal bovine serum (FBS), trypsin-EDTA, penicillin-streptomycin (penicillin 10,000 U/ml + streptomycin 10,000 μg/ml, PS), and charcoal-stripped FBS (CD-FBS) were obtained from Gibco (USA). 3-(4,5-Dimethylthiazol-2-yl)-2,5-diphenyltetrazolium bromide (MTT) was purchased from Invitrogen (USA). 17β-Estradiol (E2) and ICI 182780 (ICI, fulvestrant) were obtained from Sigma-Aldrich (USA). The Aurum Total RNA Mini Kit, iScript cDNA Synthesis Kit, and SsoAdvanced Universal SYBR Green Supermix were purchased from Bio-Rad (USA). AmpMaster 2X Taq Master Mix was obtained from GeneAll (Republic of Korea), and RedSafe Nucleic Acid Staining Solution was purchased from iNtRON Biotechnology (Republic of Korea).

### HDB7040 Preparation and Quantification

*B. breve* HDB7040 was isolated from the feces of a healthy, breast-feeding infant by HDB Cell Bank (HYUNDAI BIOLAND Co., Ltd., Republic of Korea) and preserved in the Korean Culture Center of Microorganisms (KCCM13304P). The strain was cultivated at 37°C for 24 h in glucose, yeast extract, and soy peptone-based culture medium and heated at 95°C for 1 h. The heat-treated culture was dried and kept at room temperature until further use.

For HDB7040 quantification, about 1 g of heat-killed HDB7040 powder was dissolved in a 100 ml solution and diluted at a ratio of 1:5000. The heat-killed cells in the diluted solution were observed using a Neubauer counting chamber (Marienfeld-Superior, Germany). Cells in 16 smaller squares were counted, and the total number of heat-killed HDB7040 was calculated using the following equations:

$$\text{Total number of cells (cells/g) =}\frac{\text{Total number of cells in one square}}{\text{Counted surface }(mm^{2})\times \text{Chamber depth }(mm)}\times 10^{3}(mm^{3}/g)\times \text{Dilution factor}$$

### Cell Culture

The human ER-positive breast adenocarcinoma MCF-7cells were obtained from the Korean Cell Line Bank (KCLB, Republic of Korea) and maintained in an RPMI1640 medium supplemented with 10% FBS and 1% PS in a humidified 5% CO_2_ atmosphere at 37°C. For E-screen assay and gene expression analysis, MCF-7 cells were cultured in an estrogen-free (EF) medium, Phenol Red-free RPMI-1640 with 10% CD-FBS and 1% PS.

### Cytotoxicity Assay

MCF-7 cells were seeded onto 96-well cell culture plates (1 × 10^4^ cells/well), incubated for 24 h, and treated with increasing concentrations (0-1,000 μg/ml) of HDB7040 for 24 h. After removing the culture supernatant, 100 μl of MTT solution (1 mg/ml) was added to each well, and the plates were incubated at 37°C. The formazan was solubilized using DMSO, and the absorbance was read at 570 nm using a microplate reader (Tecan, Switzerland).

### E-Screen Assay

MCF-7 cells were cultured in 96-well cell culture plates (5 × 10^3^ cells/well) for 24 h. The culture medium was replaced with an EF-medium following PBS washing, and cells were further incubated for 48 h at 37°C to induce the EF condition. MCF-7 cells were exposed to various concentrations (0-1,000 μg/ml) of HDB7040 for 144 h, and the treatment was repeated every two days. Cell proliferation was assessed by the MTT assay described above, with 1 nM of E2 as a positive control.

### ER Antagonist Study

To ensure that HDB7040 regulated the estrogen signaling pathway via ERs, the well-known ER antagonist ICI was applied to MCF-7 cells in the absence or presence of HDB7040. MCF-7 cells were cotreated with 100 nM of ICI and HDB7040 for the E-screen assay, followed by pretreatment with 100 nM of ICI for 30 min before HDB7040 treatment for RNA expression analysis.

### Animal Study

The animal experiment protocols in this study were approved by Institutional Animal Care and Use Committee of the Dt&CRO Efficacy Evaluation Center (Approval no. DTE230007; Republic of Korea). Efforts were made to minimize the number of rats used. Five-week-old SD female rats were purchased from Samtako (Republic of Korea) and housed under controlled temperature (22 ± 3°C) on a 12/12-h light-dark cycle. All rats were provided a commercial diet and water ad libitum and allowed to acclimatize for one week before ovariectomy (OVX).

Rats were anesthetized with isoflurane, subjected to Sham or OVX surgery, and divided into five groups of eight rats each (*n* = 8/group): Sham, OVX, OVX + low HDB7040 dose (7040-L, 1 × 10^9^ cells/kg), OVX + high HDB7040 dose (7040-H, 2 × 10^9^ cells/kg), and OVX + 17β-estradiol (E2, 30 μg/kg). Rats in the 7040-L and 7040-H groups were orally administered with HDB7040, whereas rats in the E2 group were intraperitoneally subjected to 17β-estradiol for eight weeks. Rats in Sham and OVX groups were instead subjected to PBS and olive oil, which were used as excipients for the HDB7040 and 17β-estradiol preparations. Body weight and food intake were measured once and twice weekly.

After sacrificing rats following anesthesia with isoflurane, the abdominal aorta serum, right femur, uteri, and visceral adipose tissues were collected analyzed. Blood concentrations of total cholesterol (TC), low-density lipoprotein cholesterol (LDL), high-density lipoprotein cholesterol (HDL), triglyceride (TG), alkaline phosphatase (ALP), bone ALP (bALP), and estradiol were analyzed using the appropriate ELISA assay kits according to the manufacturer’s instructions. Femoral trabecular bone analysis was performed by scanning the obtained femur using a vivaCT80 (Scanco Medical, Switzerland). Trabecular morphometric parameters, including bone mineral density (BMD), bone volume per tissue volume (BV/TV), trabecular number (Tb.N), and trabecular separation (Tb.Sp) were evaluated. Uteri and visceral adipose tissues were weighed. Uteri were further stained with hematoxylin and eosin (H&E), and the uteri and endometrium lengths were measured.

### Relative mRNA Expression Level Accessed by RT-PCR

MCF-7 cells were induced by the abovementioned EF condition and treated with HDB7040 (0-1,000 μg/ml) and 1 nM of E2 for 24 h. Uterus tissues were ground with a tissue homogenizer before RNA extraction. Cells or tissues were lysed, and total RNA was extracted using an Aurum Total RNA Mini Kit. The RNA concentrations were measured using a NanoDrop 2000 Spectrophotometer (Thermo Fisher Scientific, USA), and the complementary DNA (cDNA) was synthesized using 1 μg of RNA and the iScript cDNA Synthesis Kit. For real-time PCR, cDNA was amplified with specific primers using the SsoAdvanced SYBR Green Supermix, and the target gene’s relative expression was calculated according to the 2^-ΔΔCt^ equation. For RT-PCR, an AmpMasterTM 2X Taq Master Mix was used, and the PCR products were separated on a 1.5% agarose gel stained with RedSafe Nucleic Acid Staining Solution and visualized using a UV transilluminator (Bio-Rad, USA). The primer sequences used in this study are listed in Table 1.

### Statistical Analysis

Each experiment was independently performed in triplicate, and data are presented as the means ± SEM. The differences between at least three experimental groups were analyzed by one-way ANOVA followed by Dunnett’s multiple comparison test using GraphPad Prism software. The differences between the means of the two groups were analyzed by unpaired Student’s *t*-test. *p* < 0.05 indicated statistical significance.

## Results

### Total Heat-Killed HDB7040 Cell Counts

Heat-killed HDB7040 cells were counted using a counting chamber, and the result was 3.2 × 10^10^ cells/g.

### Cytotoxicity of HDB7040 Against MCF-7 Cells

**Fig. 1:**
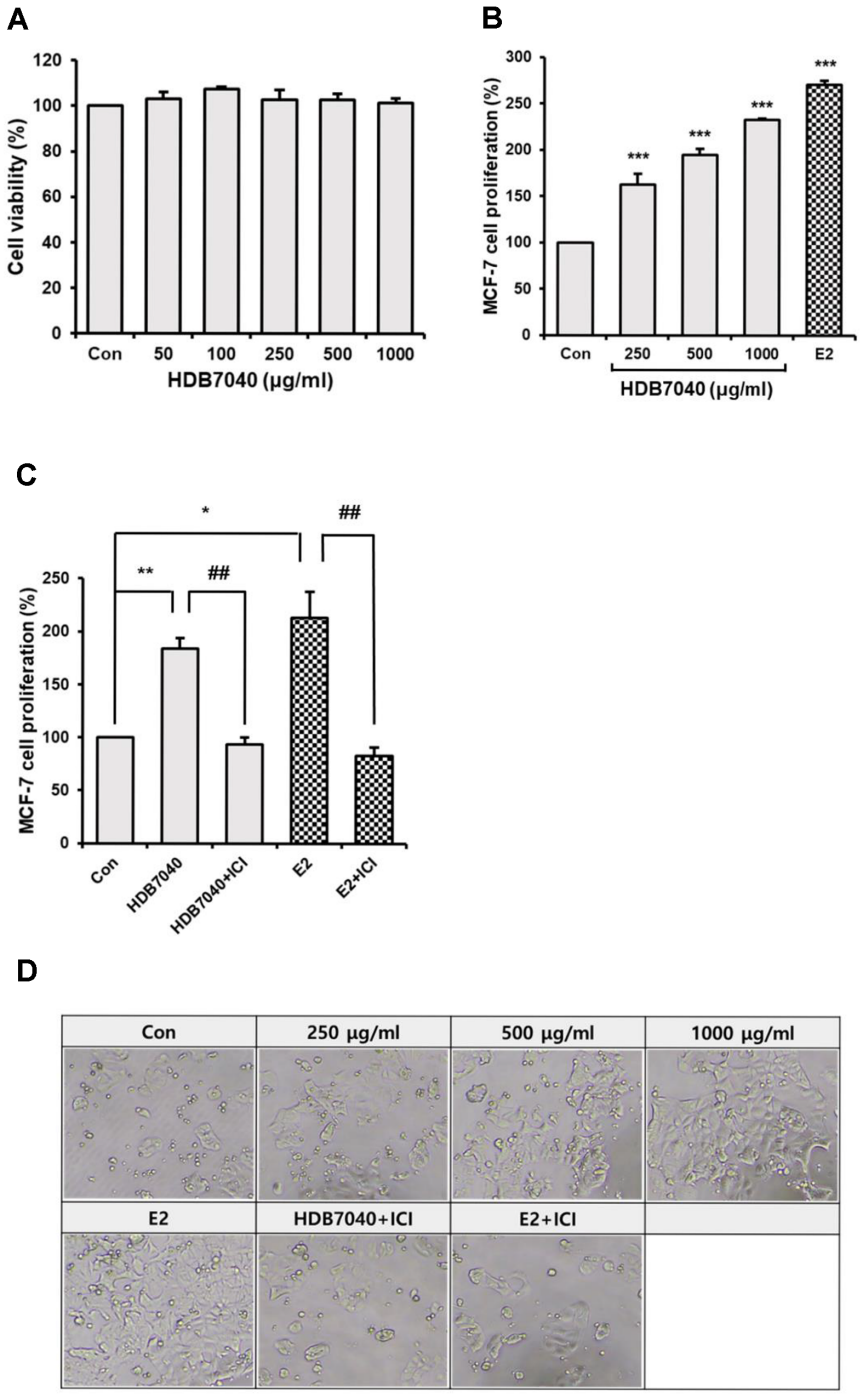
Effect of HDB7040 on MCF-7 cell viability and proliferation under EF conditions. (**A**) MCF-7 cells were treated with various concentrations of HDB7040 (0-1,000 μg/ml) for 24 h, and cell viability was investigated by MTT assay. (**B**) HDB7040-induced MCF-7 cell proliferation upon EF condition was assessed by E-screen assay, and (**C**) cellular morphology was monitored using a light microscope. (**D**) The effect of HDB7040 on ER activation was confirmed by an E-screen assay using an ER antagonist. 17β-estradiol (E2) was used as a positive control. Data are presented as the mean ± SEM (*n* = 3). *, *p* < 0.05; **, *p* < 0.01; ***, *p* < 0.001, compared to the control; ##, *p* < 0.01, compared to the non-ICI-treated group.

An MTT assay was performed to investigate whether HDB7040 has a deleterious effect on MCF-7 cell viability. HDB7040 did not have any detrimental effect on the MCF-7 cell viability up to 1,000 μg/ml (Fig. 1A). Concordantly, 1,000 μg/ml was selected as the maximum concentration for further experiments.

### HDB7040-Induced MCF-7 Cell Proliferation upon EF Condition

Since MCF-7 cell proliferation is induced through ER activation, MCF-7 cells were exposed to EF condition followed by HDB7040 treatment, and cell proliferation was assessed by MTT assay. Upon EF condition, HDB7040 remarkably and significantly promoted MCF-7 cell proliferation up to 232.9% compared to the control in a dose-dependent manner, and its effect was similar to E2 treatment (Fig. 1B and 1D). Interestingly, HDB7040-induced MCF-7 cell proliferation was significantly inhibited by 93.4% with ICI 182780 treatment (Fig. 1C and 1D). These results indicate that HDB7040 promotes MCF-7 cell proliferation via ER activation.

### HDB7040 Effect on *ESR1* and *ESR2* Expression in EF-Induced MCF-7 Cells

**Fig. 2:**
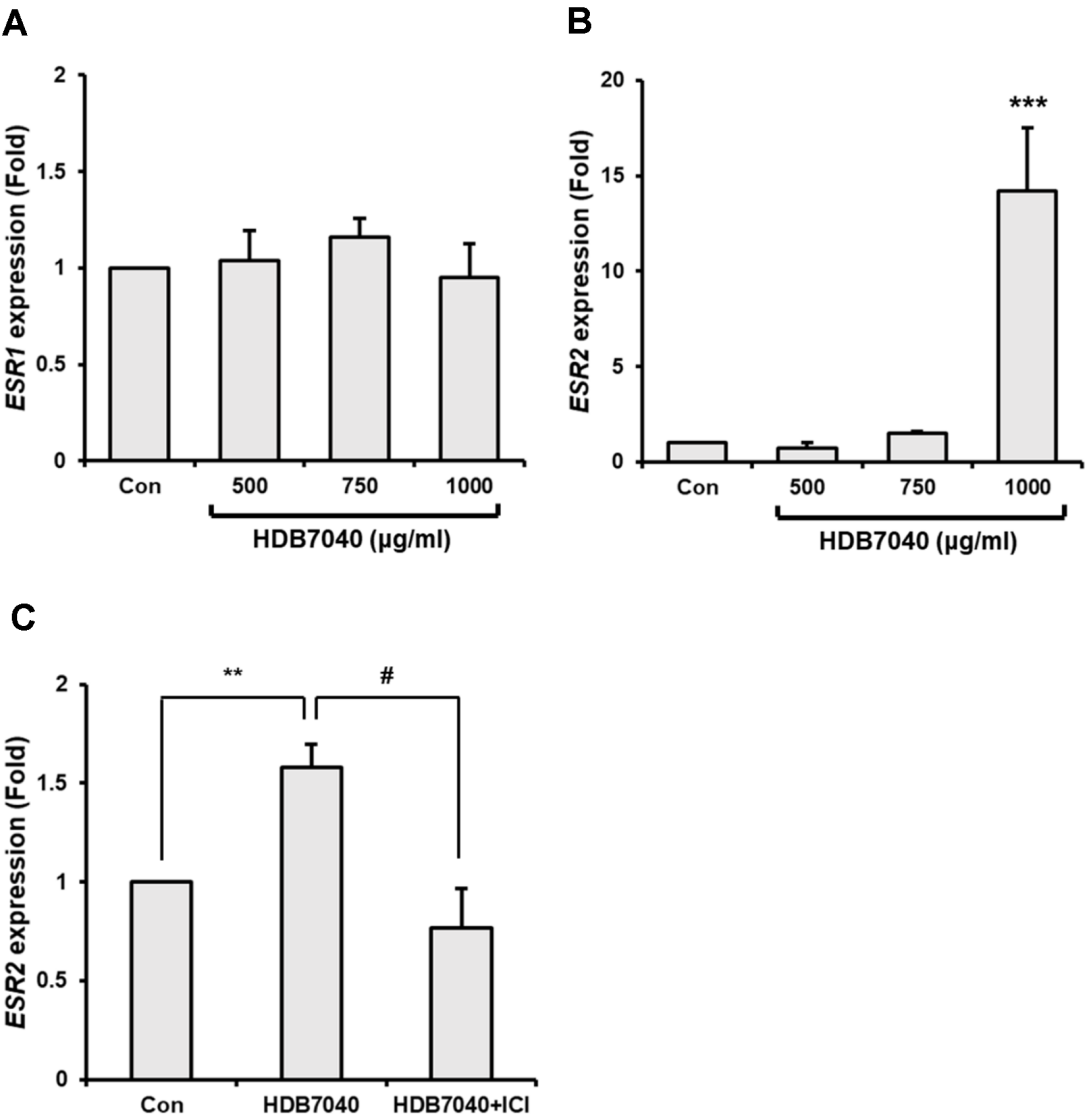
Regulatory effect of HDB7040 on ER mRNA expression. (**A**) Expression of *ESR1*, (**B**) *ESR2*, and (**C**) changes in *ESR2* expression by ICI pretreatment in HDB7040-treated MCF-7 cells expressed as a ratio to the internal standard (18S rRNA). Data are presented as the mean ± SEM (*n* = 3). **, *p* < 0.01; ***, *p* < 0.01, compared to the control; #, *p* < 0.05, compared to the non-ICI-treated group.

HDB7040 markedly and specifically stimulated *ESR2* expression in MCF-7 cells up to 14.2-fold compared to the control, but did not significantly affect *ESR1* expression (Fig. 2A and 2B). Moreover, similar to the E-screen assay, HDB7040-induced *ESR2* expression in MCF-7 cells was significantly inhibited by ICI (Fig. 2C). These results suggest that HDB7040 is involved in the specific regulation of *ESR2*, but not *ESR1*, in EF-induced MCF-7 cells.

### HDB7040 Improves Menopausal Lipid Metabolic Dysfunction in OVX Rat Models

**Fig. 3:**
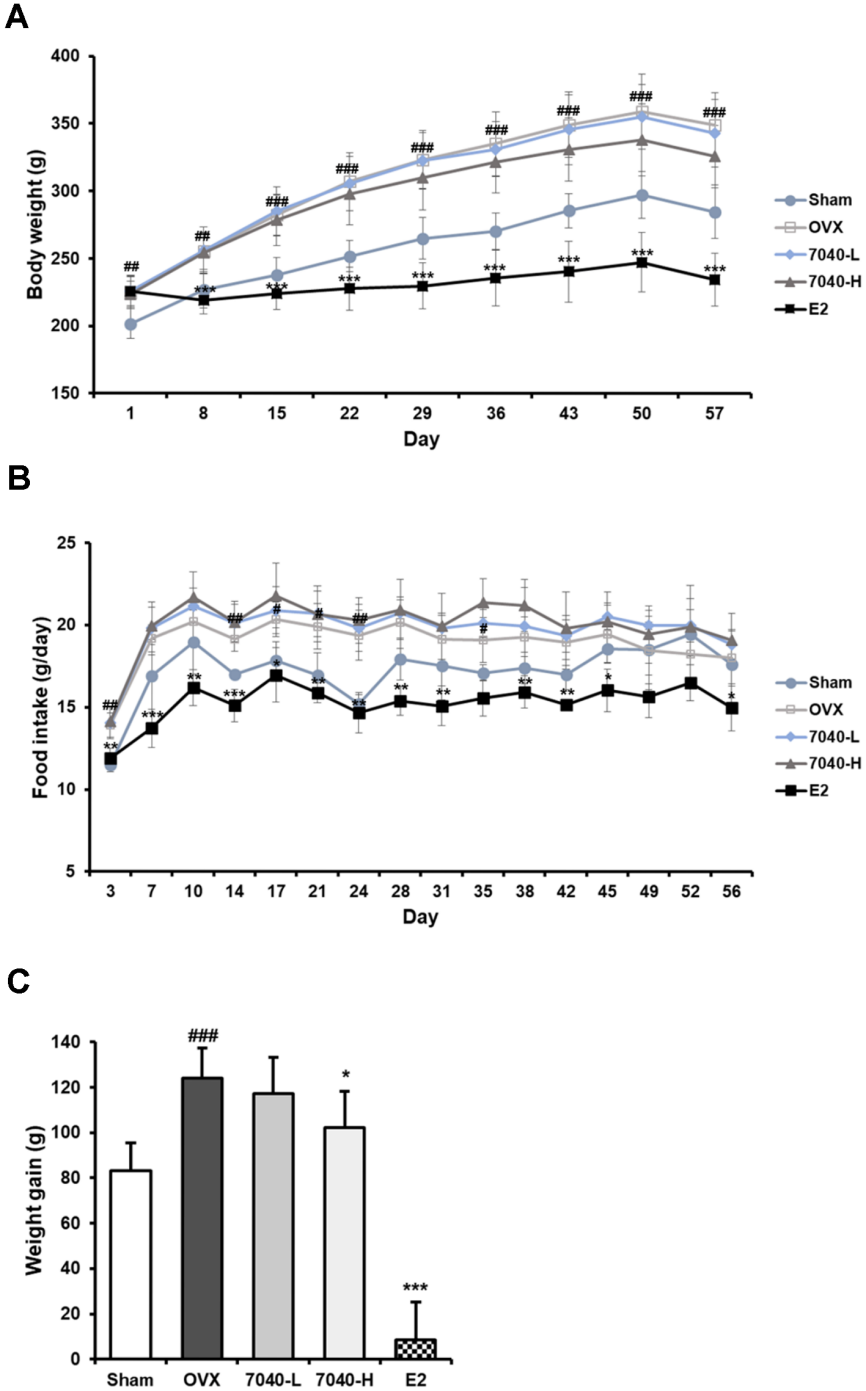
Effect of HDB7040 on the body weight change and food intake of OVX rats. SD female rats underwent OVX surgery and were orally administered with HDB7040 for eight weeks. (**A**) Body weight change and (**B**) food intake were measured once and twice a week, and (**C**) weight gain was calculated by subtracting initial body weight from final body weight. Data are presented as the mean ± SD (*n* = 8). ##, *p* < 0.01; ###, *p* < 0.001, compared to the Sham group; *, *p* < 0.05; **, *p* < 0.01; ***, *p* < 0.001, compared to the OVX group.

Compared to the Sham group, rats in the OVX group displayed significant weight gain, markedly inhibited by the E2 injection (Fig. 3A and 3C). HDB7040 slightly decreased body weight compared to OVX in a dose-dependent manner, and total weight gain was significantly inhibited in the 7040-H group by 102.2 g compared to the OVX group (124.2 g) (Fig. 3A and 3C). Meanwhile, food intake in HDB7040-administered groups did not display any significant change compared to the OVX group, whereas E2-injected groups presented the lowest food intake compared to the OVX and Sham groups (Fig. 3B).

**Fig. 4:**
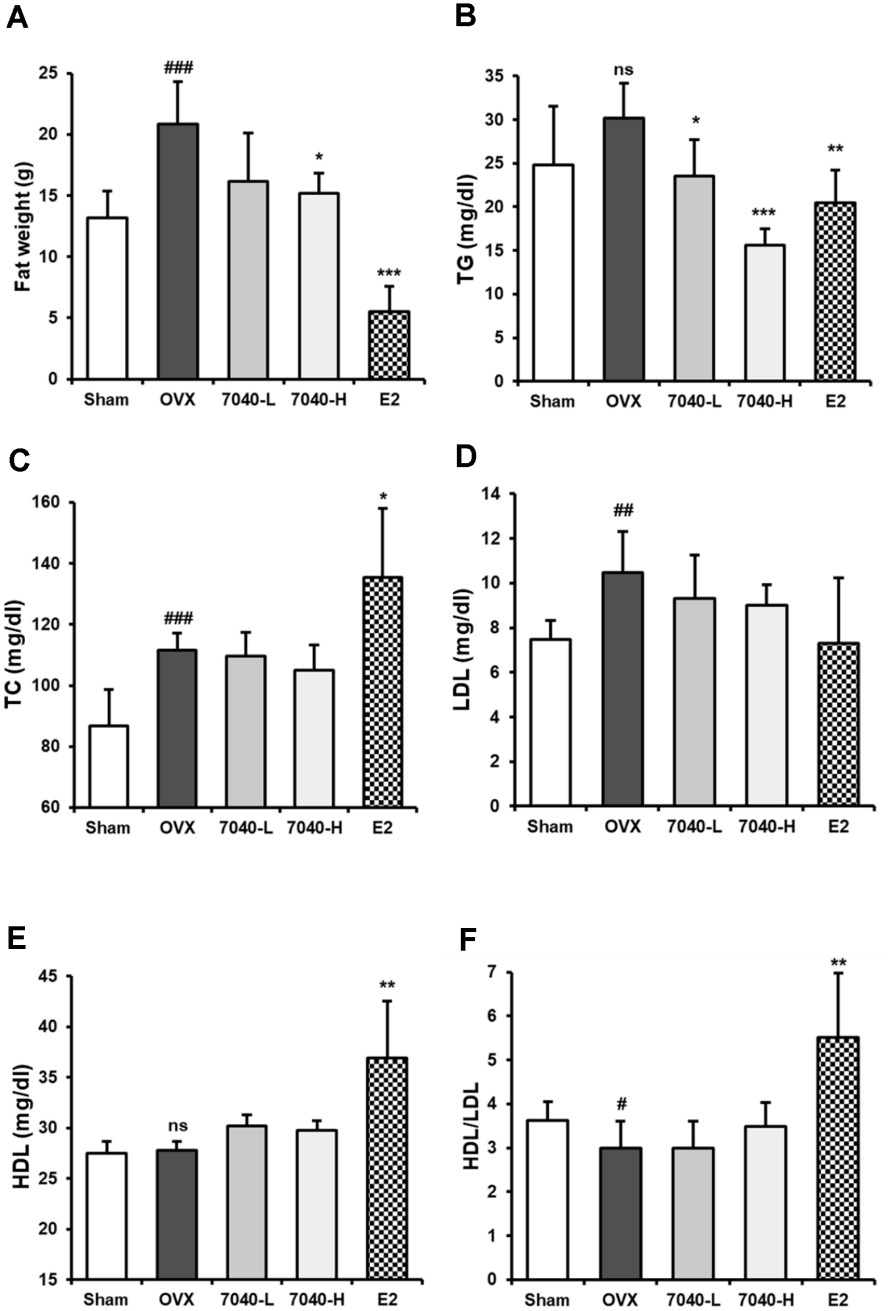
Effect of HDB7040 on the fat weight and blood concentrations of lipid metabolism biomarkers in OVX rats. After sacrifice, (**A**) visceral adipose tissues were weighed, and (**B**) blood TG, (**C**) TC, (**D**) LDL, and (**E**) HDL concentrations were measured using ELISA kits. (**F**) HDL/LDL ratio was also calculated by dividing HDL concentrations by LDL concentrations. Data are presented as the mean ± SD (*n* = 8). ##, *p* < 0.01; ###, *p* < 0.001, compared to the Sham group; *, *p* < 0.05; **, *p* < 0.01; ***, *p* < 0.001, compared to the OVX group.

Furthermore, HDB7040 significantly decreased OVX-induced fat weight (Fig. 4A) and blood TG concentration (Fig. 4B) by 15.2 g (OVX: 20.8 g) and 15.6 mg/dl (OVX: 30.2 mg/dl), respectively, in a dose-dependent manner. Blood TC (Fig. 4C) and LDL (Fig. 4D) concentrations were also increased in the OVX group compared to the Sham group by up to 111.6 and 10.48 mg/dl, respectively. HDB7040 slightly inhibited these increases by 105 and 9.0 mg/dl, respectively. OVX had no significant effect on the blood HDL concentration (Fig. 4E), but the HDL/LDL ratio displayed a significant change in the OVX group compared to the Sham group, and HDB7040 slightly recovered up to 3.5 compared to the OVX group (3.0) (Fig. 4F). These results suggest that HDB7040 suppresses menopausal weight gain and balances lipid metabolism homeostasis in the OVX rat model.

### HDB7040-Induced Bone Recovery in OVX Rat Models

**Fig. 5:**
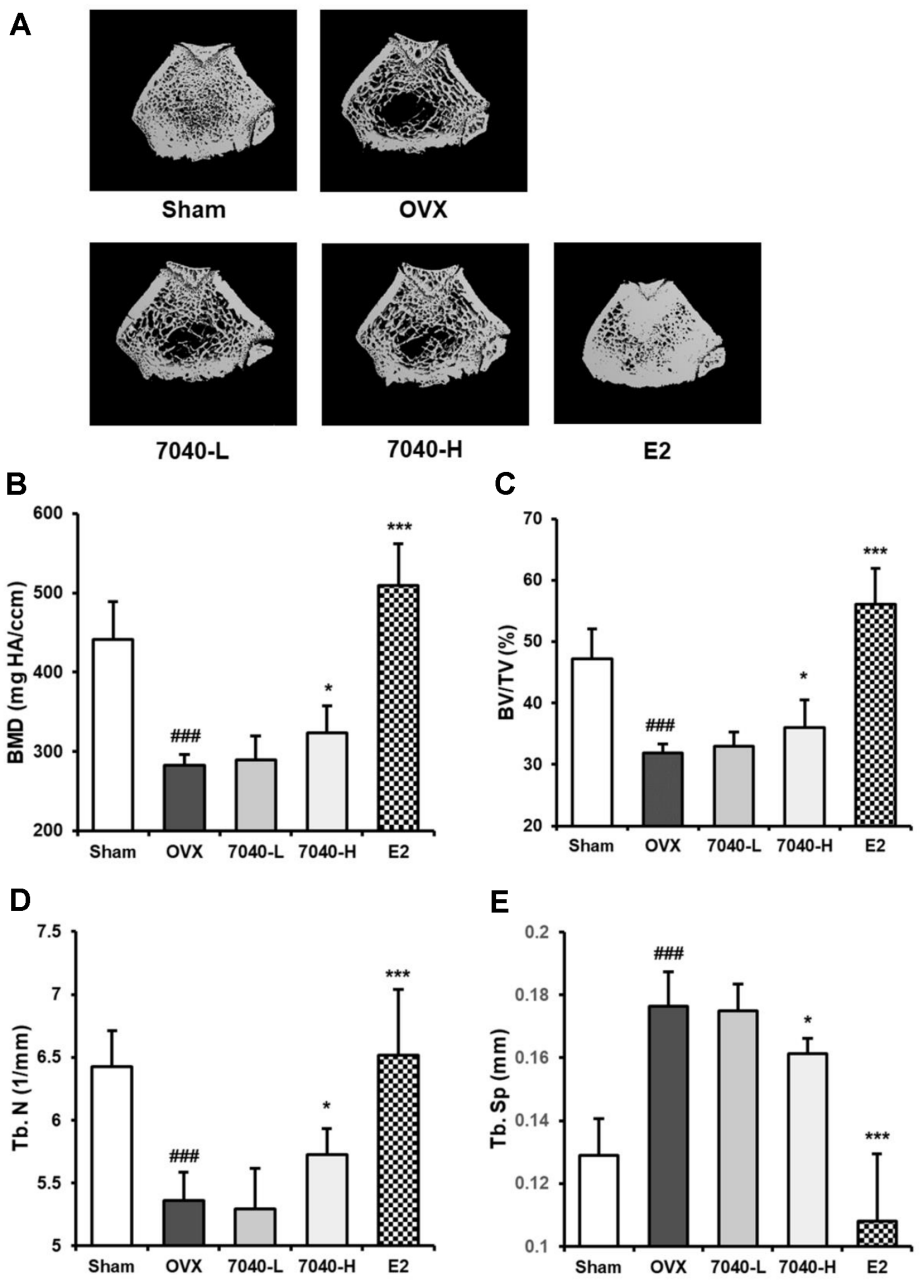
Inhibitory effect of HDB7040 on OVX-induced bone loss. (**A**) Right femora collected from rats were scanned using a μCT scanner, and femoral trabecular analytic markers were evaluated, including (**B**) BMD, (**C**) BV/TV, (**D**) Tb.N, and (**E**) Tb.Sp. Data are presented as the mean ± SD (*n* = 8). ###, *p* < 0.001, compared to the Sham group; *, *p* < 0.05; ***, *p* < 0.001, compared to the OVX group.

Estrogen deficiency causes osteoporotic trabecular microstructure collapse in OVX rodents accompanied by altered trabecular morphometric parameters, such as BMD, BV/TV, Tb.N, and Tb.Sp [14]. The microstructure of the distal femur in the OVX groups was destructed compared to the Sham group (Fig. 5A), and administrating HDB7040 markedly restored the estrogen deficiency-induced trabecular bone loss in a dose-dependent manner. Accordingly, BMD (Fig. 5B), BV/TV (Fig. 5C), and Tb.N (Fig. 5D) were significantly decreased in the OVX groups compared to the Sham group and remarkably alleviated in HDB7040-administered groups. Furthermore, administrating HDB7040 significantly reduced the OVX-induced Tb.Sp increase in a dose-dependent manner (Fig. 5E). These results demonstrate that HDB7040 restores estrogen deficiency-induced bone loss in menopausal rats.

### HDB7040 Normalizes OVX-Induced Blood ALP Levels in OVX Rat Models

**Fig. 6:**
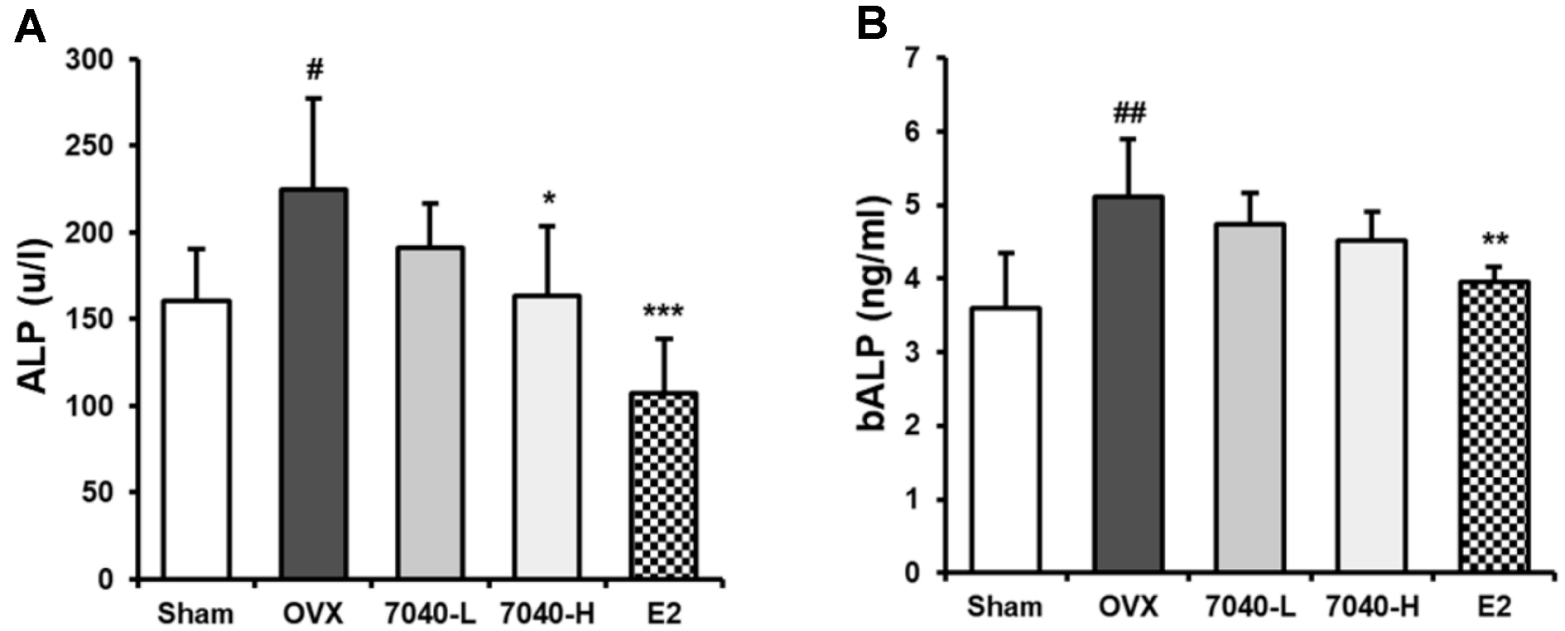
Effect of HDB7040 on serum ALP levels in OVX rats. (**A**) ALP and (**B**) bALP levels in serum were assessed using ELISA kits. Data are presented as the mean ± SD (*n* = 8). #, *p* < 0.05; ##, *p* < 0.01, compared to the Sham group; *, *p* < 0.05; **, *p* < 0.01; ***, *p* < 0.001, compared to the OVX group.

The inhibitory effect of HDB7040 on the OVX-induced bone loss was further assessed by measuring blood ALP levels. Total ALP activity (Fig. 6A) and bALP (Fig. 6B) concentration were markedly increased in the OVX groups up to 224.7 u/l and 5.12 ng/ml, respectively. HDB7040 inhibited the OVX-induced ALP increase by 163.1 u/l and 4.5 ng/ml, respectively, similar to E2. These results show that HDB7040 regulates ALP levels and secretions, improving OVX-induced osteoporosis.

### HDB7040 Effect on Uterine Growth and Female Hormone in OVX Rat Models

**Fig. 7:**
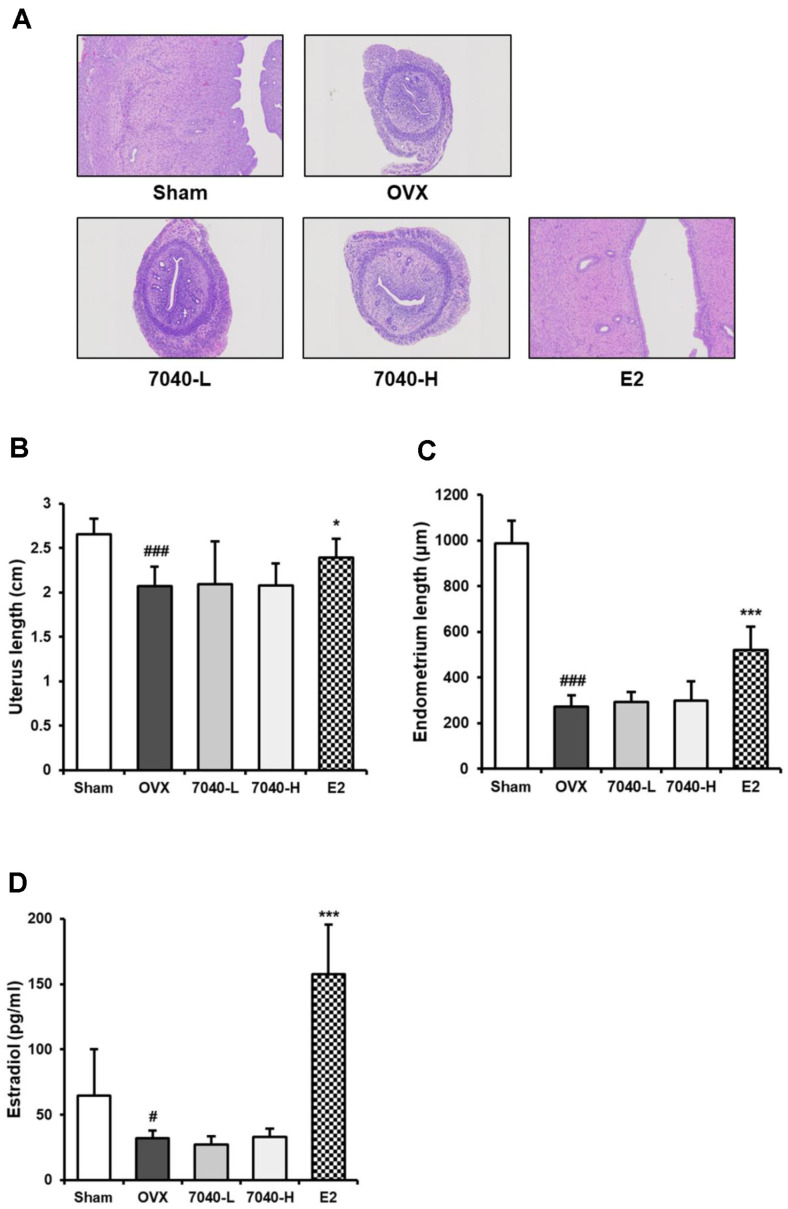
Effect of HDB7040 on uterine histology and blood estradiol levels in OVX rats. (**A**) Uteri tissues were stained, and their histological changes were monitored by H&E staining. (**B**) Uterus and (**C**) endometrium lengths were measured, and (**D**) estradiol blood concentrations were evaluated by ELISA assay. Data are presented as the mean ± SD (*n* = 8). #, *p* < 0.05; ###, *p* < 0.001, compared to the Sham group; *, *p* < 0.05; ***, *p* < 0.001, compared to the OVX group.

Uterus and endometrium lengths in the OVX group were markedly decreased by 2.07 cm and 270.6 μm, respectively, compared to the Sham group (2.65 cm and 988.1 μm) (Fig. 7A-7C). The E2 injection significantly recovered uterus and endometrium lengths up to 2.39 cm and 521.4 μm, respectively, whereas HDB7040 had no significant influence on the uterus (2.07 cm) and endometrium (298.5 μm). Similarly, blood estradiol concentration was decreased in the OVX group by 31.9 pg/ml compared to the Sham group (64.5 pg/ml), and significantly increased in the E2 group up to 157.8 pg/ml; however, administrating HDB7040 maintained blood estradiol concentration at a level (32.8 pg/ml) similar to that of the OVX group (Fig. 7D). These results demonstrate that HDB7040 effectively attenuates various menopausal symptoms, including lipid metabolism abnormalities and osteoporosis, in a similar manner to that observed in the E2-injected group. However, unlike E2, HDB7040 does not significantly affect uterine growth and blood estradiol concentrations.

### Regulatory Effect of HDB7040 on Estrogen-Related Gene Expression in OVX Rat Uteri

**Fig. 8:**
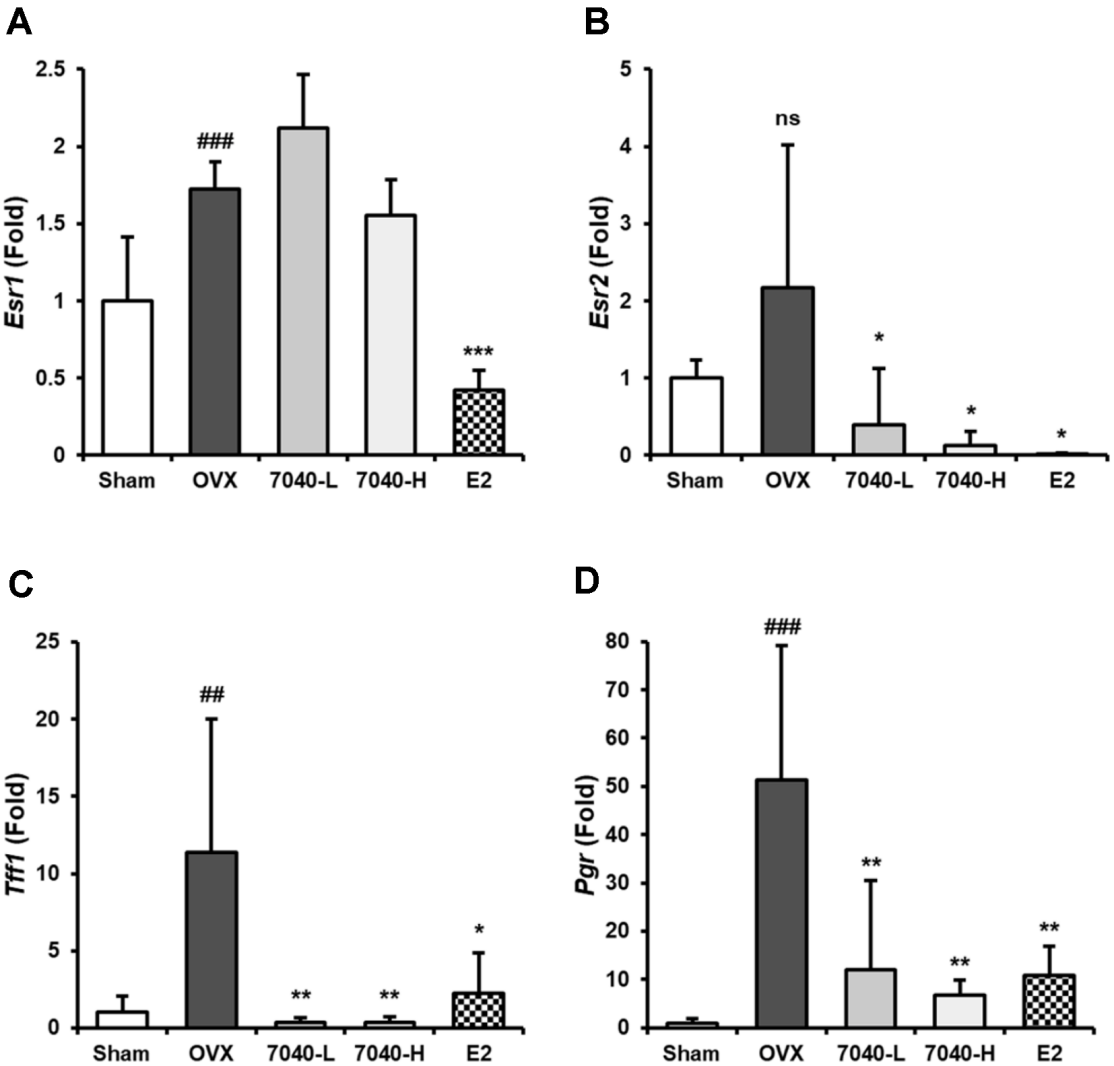
Regulatory effect of HDB7040 on ER-related gene expression in OVX rat uteri. The expression of (**A**) *Esr1*, (**B**) *Esr2*, (**C**) *Tff1*, and (**D**) *Pgr* in the uteri were assessed by real-time PCR and expressed as a ratio to the internal standard (β- actin). Data are presented as the mean ± SD (*n* = 8). ##, *p* < 0.01; ###, *p* < 0.001, compared to the Sham group; *, *p* < 0.05; **, *p* < 0.01; ***, *p* < 0.001, compared to the OVX group.

To investigate the effect of HDB7040 on estrogen-related gene expressions in OVX rats, uteri were homogenized and subjected to qPCR analysis. The OVX-induced expression of *Esr1* and *Esr2* in uteri increased up to 1.7- and 2.2-fold, respectively, compared to the Sham group, while expression was significantly downregulated after E2 treatment by 0.4- and 0.02-fold, respectively (Fig. 8A and 8B). Interestingly, HDB7040 significantly suppressed the expression of only *Esr2* by 0.1-fold compared to the OVX group (Fig. 8B). Furthermore, HDB7040 also markedly downregulated OVX-induced *Tff1* (11.4- fold) and *Pgr* (51.3- fold) expression by 0.3- and 6.8-fold, respectively, similar to E2 (Fig. 8C and 8D) These results suggest that HDB7040 has anti-menopausal activity via the specific regulation of *Esr2* and the estrogen-related genes, *Tff1* and *Pgr*.

## Discussion

This study focused on the attenuating effect of the novel, heat-killed *B. breve* HDB7040 on menopausal symptoms. MCF-7 cells express diverse sex hormone receptors, including ERs, and their proliferation depends on the activation of these receptors [15]. No research has ever suggested a facilitative effect of microbes on MCF-7 cell proliferation under EF conditions; however, anti-menopausal botanical extracts and phytochemicals can promote MCF-7 cell proliferation in EF conditions [16-19]. Agreeing with previous studies, our results indicated that HDB7040 remarkably induces MCF-7 cell proliferation in EF conditions. Furthermore, this proliferation is entirely inhibited by the ER antagonist, ICI. The latter interrupts ER dimerization impairing estrogen-related gene transcription [20]; therefore, the candidates expected to activate ER signaling pathways cannot promote MCF-7 proliferation in the presence of ICI. Similar to our study, previous studies also suggested that MCF-7 cell proliferation stimulated by novel anti-menopausal substances was blocked by ICI [16-19]. Our study indicates that HDB7040 stimulates the ER-mediated signaling pathway.[Fig F9]

ER modulators are mainly selective for ERα and often provoke serious illnesses, including reproductive and cardiovascular diseases [5, 6]. ERβ-specific modulators are considered safer than ERα-activator or non-specific estrogens since they rarely activate ERα, thus the growing necessity to develop ERβ-specific modulators for safe menopause treatment [6, 21]. This study demonstrated that HDB7040 only stimulated *ESR2* expression, not *ESR1*, in EF-induced MCF-7 cells, which was inhibited by ICI, similar to the E-screen assay results. *Agrimonia pilosa* extracts exert anti-menopausal activity via binding ERα and ERβ [17], and Lee *et al*. also indicated that soybean germ extract and *L. gasseri* LGA1 upregulate *ESR1* and *ESR2* expression in MCF-7 cells [22]. In contrast, HDB7040 exhibited a specific regulatory effect on *ESR2* expression, but not on that of *ESR1*.

HDB7040 effectively reduced estrogen deficiency-induced weight gain, fat mass, blood TG, TC, and LDL-levels and recovered HDL/LDL in OVX rats. Estrogen deficiency during menopause provokes metabolic dysfunction, resulting in weight gain and abnormal lipid metabolism [6]. Many researchers have suggested that administrating health-improving microbes could reverse menopausal metabolic disorders. Similar to our study, Lim *et al*. reported that administrating *L. acidophilus* YT1 orally decreased OVX-induced body weight gain and fat mass in OVX rats [23]. Myeong *et al*. have also demonstrated that heat-killed *L. plantarum* MD35 inhibited weight gain in OVX mice [13]. Additionally, *L. intestinalis* and *L. paracasei* were effective against menopausal obesity in OVX rodent models [24. 25].

Osteoporosis is one of the hallmarks of menopause [1], and several authors have suggested that supplementing beneficial microbes could mitigate menopausal osteoporosis. Guo et al. have demonstrated that *L. rhamnosus* GG attenuated OVX-induced osteoporosis by reversing diminished femur microstructure in OVX rats [26]. Yu *et al*. have also reported that *L. brevis* ameliorated osteoporosis by reducing the elevated blood ALP levels and improving trabecular parameters in OVX rats [27]. *L. reuteri* and CaF_2_ nanoparticles displayed anti-osteoporotic activity in OVX rats by stimulating tibia and femoral growth and decreasing OVX-induced blood ALP levels [28]. In our study, HDB7040 exerted a similar anti-osteoporotic activity against OVX-induced bone loss by improving trabecular morphometric parameters, BMD, BV/TV, Tb.N, and Tb.Sp, and diminishing elevated ALP levels in OVX rats. ALP is a typical osteoblastic marker used in osteoporosis diagnosis, and abnormally elevated ALP levels can denote bone fracture or low BMD in menopausal females in clinics [29, 30]. Our study demonstrated that HDB7040 efficiently mended estrogen deficiency-induced menopausal osteoporosis.

Menopausal symptoms can be improved by hormone treatment, which can cause uterine hypertrophy and cancer [1, 3]. Thus, HDB7040-induced attenuation of OVX-induced symptoms, which did not significantly affect uterine atrophy and blood estradiol levels, is an interesting observation. Several anti-menopausal microbes, such as *L. plantarum* MD35 and *L. rhamnosus* GG, do not influence uterine index or serum estradiol concentrations in OVX rodents [13, 26]. Our study pointed out that the anti-menopausal effect of HDB7040 hardly affected the female hormone system, meaning that it poses lower risk of incidence of female diseases.

Interestingly, the OVX-induced expression of *Esr2*, *Tff1*, and *Pgr* in uteri were normalized in HDB7040-administered groups, similar to the E2-injected group, and it did not change *Esr1* expression. Ovariectomy enhances ER mRNA expression in rat uteri due to the binding status of steroid hormones [31, 32]. ER expression is negatively regulated by ER-regulatory substances, such as E2, due to the changes in ligand binding capacity [31. 32]. During menopause, these changes in ERβ expression are correlated with menopausal metabolic disorders and osteoporosis; therefore, regulating ERβ can be a strategy for attenuating menopausal symptoms [6, 23, 33]. Trefoil factor 1 (TFF1) is a marker of ER responsiveness and is related to tumor invasion and angiogenesis; hence its use as a diagnostic marker of various diseases, including cancer [34]. Progesterone receptor (PGR), another estrogen-responsive receptor, participates in crosstalk with ERs under hormonal stimuli, and a previous report suggested that ERβ suppresses *PGR* expression in rat uteri [35]. According to these findings, our study indicated that the HDB7040-induced specific regulation of the estrogen-related genes, *Esr2*, *Tff1*, and *Pgr*, is central in anti-menopausal activity.

Taken collectively, our study demonstrated that heat-killed *B. breve* HDB7040 exerts an anti-menopausal activity via the specific regulation of ERβ, not ERα, thereby normalizing estrogen deficiency-mediated metabolic disorders and recovering menopausal bone loss without changes in blood estradiol concentrations and uterine atrophy. Although a more definite mechanism of the action for HDB7040 must be clarified in further studies, our findings provide a novel, potent beneficial ingredient to treat menopausal syndrome, and one that also avoids the common side effects caused by menopausal therapy.

## Figures and Tables

**Fig. 9 F9:**
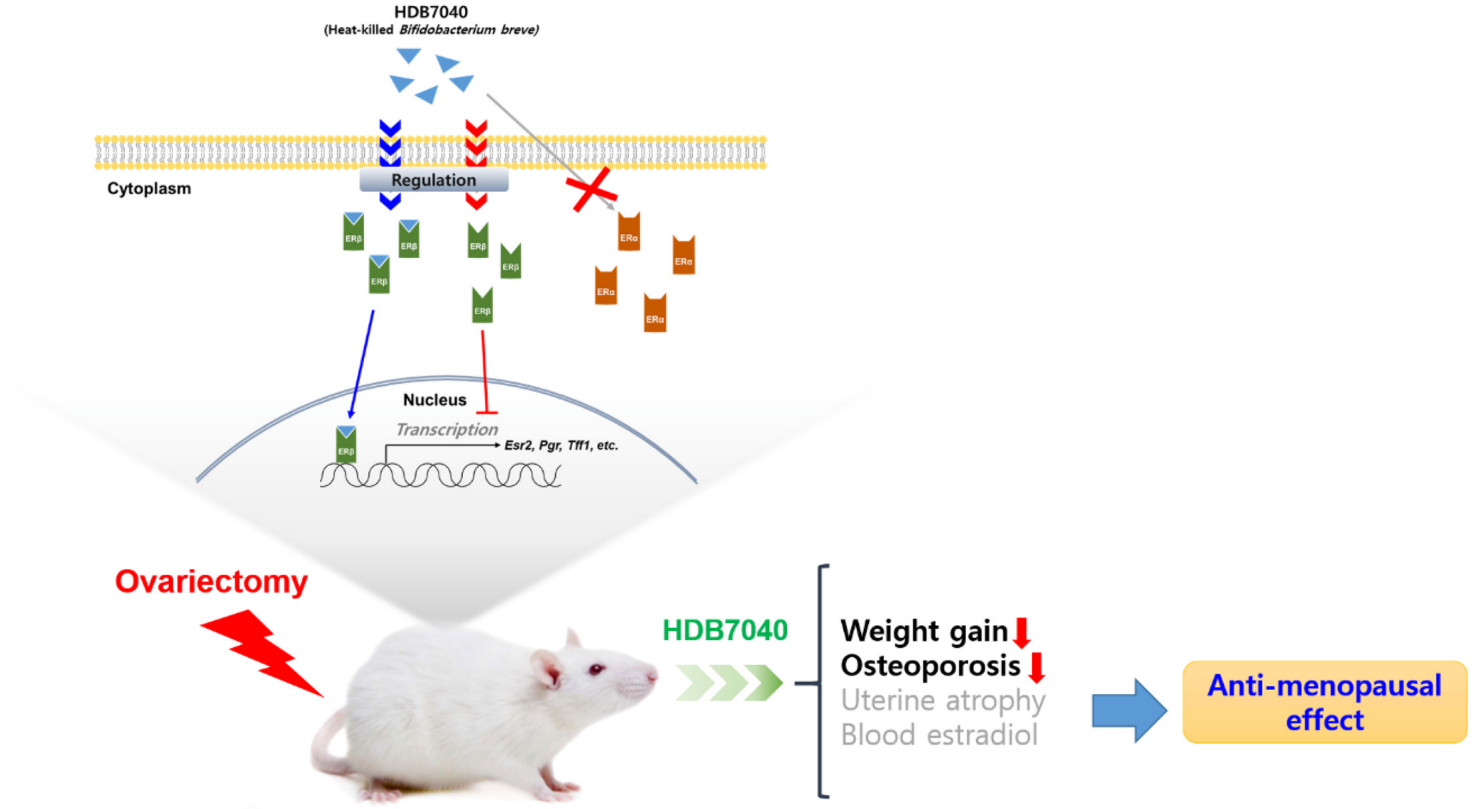
Graphical abstract. Heat-killed *B. breve* HDB7040 selectively modulates the expression of ERβ and related gene (*Pgr* and *Tff1*), alleviating various menopausal symptoms in vitro and in vivo.

**Table 1:** Primer sequences used in this study.

Origin	Gene	Primer sequences
*Homo sapiens*	*18S rRNA*	F^a^: CGG CTA CCA CAT CCA AGG AA R^b^: GCT GGA ATT ACC GCG GCT GC
	*ESR1*	F: CGA CAT GCT GCT GGC TAC ATC R: AGA CTT CAG GGT GCT GGA CAG A
	*ESR2*	F: AGC ACG GCT CCA TAT ACA TAC C R: TGG ACC ACT AAA GGA GAA AGG T
*Rattus norvegicus*	*Actinb*	F: AGT ACA ACC TTC TTG CAG CTC CT R: TGC CGG AGC CGT TGT CG
	*Esr1*	F: AGC ACA TTC CTT CCT TCC GTC R: GCC ACC CTG CTG GTT CAA A
	*Esr2*	F: TGG ATG GAG GTG CTA ATG GTG R: CCC CTC ATC CCT GTC CAG AA
	*Tff1*	F: AGG AAG AAA CAT GTG CCG TGA R: TCT CAA TGA CCA GAG GTC GGA
	*Pgr*	F: TTC CGC CAC TCA TCA ACC TG R: AAA GAG CTG GAA GTG TCG GG

^a^F, Forward; ^b^R, Reverse
